# Old Dogs and New Tricks: Assessing Idiom Knowledge Amongst Native Speakers of Different Ages

**DOI:** 10.1007/s10936-023-09996-7

**Published:** 2023-08-02

**Authors:** Gareth Carrol

**Affiliations:** Department of English Language and Linguistics, University of Birmingham, Edgbaston, Birmingham, B15 2TT UK

**Keywords:** Idioms, Formulaic language, Lifespan development, Language development, Vocabulary size

## Abstract

Idioms, along with other formulaic multiword phrases, represent a substantial part of vocabulary knowledge. This study investigates how idiom knowledge develops through the adult lifespan, comparing familiarity and transparency ratings for a large set of common English idioms. A total of 237 participants, ranging from 18 to 77 years old, collectively rated 200 idioms. They also completed a short single-word vocabulary test and provided information about their educational background. Results showed a clear increase in idiom and single-word knowledge throughout the lifespan. For idioms, this represented a jump from the youngest age-group, then a steady increase from the age of around 25 onward. Single word vocabulary knowledge increased more evenly as a function of age. Perceptions of transparency were not affected in the same way. I discuss what these results suggest about the development of vocabulary through the lifespan.

## Introduction

Vocabulary knowledge is both multifaceted, being made up of an array of words and other lexical units (phrases, idioms, collocations), and dynamic, being something that never really reaches a definitive “end point” for anyone. The development of vocabulary naturally sees its biggest growth throughout childhood, and word knowledge increases steadily as children age (Anglin, 1993; Segbers & Schroeder, 2016; Smith, 1941). Nation and Coxhead (2021) estimated growth of approximately 1000 words per year from two years old to around 15. Brysbaert et al. (2016) suggested that vocabulary levels out by around age 20, but estimated continued growth in vocabulary knowledge of approximately two new lemmas every two days, on average, up to age 60. Estimates of the typical adult vocabulary size therefore vary because of fundamental questions such as what should be counted (words, lemmas, word families), whether we are interested in receptive or productive knowledge, and at what point it can be considered that a word is “known”, and therefore can be counted as a part of any individual’s lexicon (Nation & Coxhead, 2021).

In this paper, the question of idiom knowledge is investigated, to compare lifespan development for idioms compared to broader (single-word) vocabulary knowledge. Idioms and other formulaic phrases represent an important part of “nativelike” language ability (Pawley & Syder, 1983), playing a key role in a range of communicative functions (Schmitt, 2017). Estimates vary as to the prevalence of formulaic expressions, with Jackendoff (1995) claiming that they are at least as numerous as the number of single words in English. Martinez and Schmitt (2012) developed a list of 505 frequent formulaic phrases, all of which occurred with sufficient frequency to rank amongst the 5000 most frequent English word families (which they suggest is an upper limit for high frequency vocabulary). Idioms, as one specific example of formulaic language, are particularly widespread, with Brenner (2003) estimating at least 10,000 in English, although there is considerable variation in how often individual idioms are used (Grant & Nation, 2006; Moon, 1998), and even common idioms may occur relatively few times in corpora, at least compared to individual words. Like other vocabulary, idioms also fall out of fashion and new ones emerge (Carrol, 2022), hence idiom knowledge is neither monolithic nor static.

The present study builds on the work of Sprenger et al. (2019), who conducted a similar investigation of Dutch idioms. They sampled close to 200 idioms over two studies, with participants ranging from 12 to 86 years old. Broadly, they found a clear effect of age, with idiom familiarity increasing steadily well into adulthood (they estimate around 30 years of age) before levelling off. A more modest increase was then observed up until around 55–60 years of age. They also found more variability in knowledge amongst younger participants, an effect of frequency that was more pronounced for younger participants, and a relationship between decomposability (how well the figurative and literal meanings align) and familiarity that manifested most clearly for younger participants. Taken as a whole, the results support a picture where idiom knowledge: a) lags behind single-word vocabulary knowledge in terms of “adult-like” attainment; and b) is more variable and more affected by factors such as frequency and decomposability for younger than older adults. A limited number of other studies have also found evidence pointing toward an increase in idiom knowledge over the lifespan. Kuiper et al. (2009) and Hung and Nippold (2014) both reported better performance (on a recall task and a familiarity rating/explanation task, respectively) for older than for younger participants. However, both studies are limited by a small item pool, with just 20 items per study included, so more evidence is required for us to build a full picture of how English idioms develop as language users age.

Researchers have also attempted to understand different aspects of idiom processing at different stages of the lifespan. Psycholinguistic research has demonstrated a substantial direct retrieval component for idioms, at least during the early stages of processing (Titone et al., 2019), hence knowledge of the form and meaning of phrases is a prerequisite for their use in natural language. Lack of familiarity with an idiom can cause significant disruption in processing and interpretation (Carrol & Littlemore, 2020), and it is self-evident that lack of knowledge of the form of an idiom would preclude any ability to use it productively. The ability to understand figurative language appropriately begins to resemble adult-like behaviour by the age of around 10–11 (Levorato & Cacciari, 1995, 1999; Vulchanova et al., 2011), but even before this point, children seem able to utilise general mechanisms such as guessing from context or semantic analysis to successfully infer the meaning of some idioms (Cain & Towse, 2008; Cain et al., 2009; Gibbs, 1987, 1991). Children can generally comprehend idioms earlier than they can produce them (Levorato & Cacciari, 1995), but given the relative infrequency with which individual items occur, questions remain over quite how children learn idioms in the first place. Whilst Wray (2002) and Tomasello (2003) highlight that importance of recurrent phrases in children’s acquisition of language, Wray et al. (2016) also point out that the lack of frequency for individual idioms may run counter to the fundamental ideas behind a usage-based approach to development (sufficient exposure to work out the form and also the patterns of usage). In contrast, Reuterskiöld and Van Lancker Sidtis (2012) demonstrated that children of eight years old were significantly better at recalling idioms compared to novel non-literal phrases after only one exposure, and suggested that the semantic difficulty that they pose may contribute to greater salience and therefore memorability.

At the other end of the lifespan, studies of older adults have focused on the effect that increased idiom knowledge might have on processing, or the effect of decline in other aspects of cognition. Broadly, vocabulary knowledge is generally seen to increase over time (Brysbaert et al., 2016; Keuleers et al., 2015), even if aspects such as lexical retrieval and naming tend to decline (e.g. Goral et al., 2007). For idioms, older adults show facilitation (compared to younger adults) when reading idioms, but also show greater difficulty reading sentences biasing a literal interpretation (Haeuser et al., 2021). Older adults may also be slower to make judgements of literalness for idioms (Westbury & Titone, 2011), and literal priming for older adults with low verbal fluency may be reduced (Grindrod & Raizen, 2019). On a production test, frozenness rather than familiarity predicted performance for older adults (with the reverse true for younger adults) (Hyun et al., 2014), and Coane et al. (2014) reported higher familiarity for older adults, although this did not lead to any age-related differences in their recognition and memory test. Together, these results suggest that idioms and their figurative meanings are more strongly entrenched for older adults, as a result of their greater experience with the language. Conversely, older adults performed worse than younger adults on an idiom production task involving story completion (Conner et al., 2011), which the authors took as an indication of a similar decline in lexical access as observed in single-word naming.

## Summary

The present study aims to provide an overall description of knowledge of English idioms throughout the adult lifespan, comparable to that provided by Sprenger et al. (2019) for Dutch idioms. As well as providing an age-normed set of data for British English idioms, we also aim to compare development of knowledge for idioms and single-word vocabulary, and to investigate the interplay of factors like transparency and familiarity (for idioms), and education level (for idioms and single words).

## Methodology

### Materials

Idioms were first collected from a series of previously published lists. These lists have collected normative data on a range of characteristics known to be important to how idioms are processed and understood (such as familiarity, perceived decomposability, literalness, etc.), but none have directly looked at how dimensions of idiom knowledge vary according to age, nor how idiom knowledge relates directly to vocabulary size.

The lists consulted were Titone and Connine (1994), Libben and Titone (2008), Nordmann et al. (2014), Bulkes and Tanner (2017) and Nordmann and Jambazova (2017). These lists range from 100 to 870 items, hence provided a wide range of candidates. From these, we discounted any idioms thought to be specific to American English (since the participant population would be speakers of British English), and also supplemented the list with some idioms likely to be more old-fashioned (taken from Wray et al., 2016), and some chosen to be more “modern” (taken from Carrol, 2022). A final list of 200 items was chosen to represent a range of British English idioms, all confirmed to be in (more or less) common use with a Google search. These varied in terms of their syntactic structure, from verb-determiner-noun idioms (e.g. *call the shots, bite the bullet*) to longer complex noun phrases (e.g. *a knight in shining armour, an accident waiting to happen*).

The idioms were arranged into alphabetical order and divided across five lists. No specific criteria for balancing these lists were imposed, although any idioms sharing the same initial verb were arranged on different lists (e.g. the overall set contained *go down a storm*, *go into your shell*, *go off the boil*, *go off the rails* and *go through the motions*, hence these “go” idioms all appeared on different presentation lists). Each idiom was then put into a short, neutral context (e.g. *clean up your act: He would clean up his act*), and a general meaning was assigned to each one (e.g. *clean up your act* = “start behaving in a better way”). Definitions were checked using online dictionaries, to ensure that they represented the commonly understood meaning for each phrase. Lemmatised frequencies for each item were also obtained from the NOW corpus, which provides an up-to-date monitor corpus of English usage (Davies, 2016), then converted to the Zipf scale (van Heuven et al., 2014).^1^

A vocabulary test was also prepared, based on the Vocabulary Size Test developed by Nation and Beglar (2007). This test originally sampled words from the 14,000 most frequent words in English. An updated version (available from www.wgtn.ac.nz/lals/resources/paul-nations-resources/vocabulary-tests) samples 100 words from the 20,000 most frequent words in English. As the participants would be native speakers of English, the test was adapted to start halfway through, hence only 50 words (taken from the 10,000 to 20,000 most frequent in English) were included. This would provide an indication of variation in vocabulary size amongst participants.

### Participants

Participants were recruited via Prolific (www.prolific.co) and paid £4 for their responses (which worked out at an hourly rate of £8–10 for most participants). The screening criteria were set to limit participants to those based in the UK whose first language was English. The age range was set to recruit respondents from 18 to 80 (the oldest respondent was 77), with subsequent versions of the survey relaunched to target smaller age brackets, to ensure that participants of all ages were collected. A total of 237 responses were received, with Prolific set to collect an approximately equal number of male and female respondents. Participants were randomly assigned to one of the five presentation lists, meaning that each idiom was rated by a minimum of 46 people.

### Procedure

Idioms were presented in an online survey which began by describing the research and asking participants for their consent to take part. An explanation of the idiom rating and vocabulary test were then given, with examples for each. Idioms were presented in isolation, then in a neutral example sentence, and participants were asked to rate on a 5-point scale how familiar they were with the phrase as an idiom. Here, “1” would represent a phrase that a participant had never heard before, and “5” would represent a phrase that a participant knew very well. Participants were told that number in-between could be used for less well-known phrases, for instance if they recognised a phrase but didn’t have full knowledge of what it meant, they might choose “3”.

On the following screen each phrase was then presented with its meaning. Participants were first asked whether this was the meaning they thought of for this phrase, yes or no. This would provide a further indication of familiarity, as a participant may have indicated that they were familiar with a phrase but subsequently have discovered that they didn’t actually know the meaning. Participants were then asked (with the phrase and its meaning still visible) to indicate how transparent they thought the phrase was, explained as how easily they could guess the meaning from the component words if they had never heard it before. Here, transparency is functionally equivalent to what other studies have called decomposability, since it explicitly asks participants to consider the meaning of each idiom as it relates to the component words. (See Carrol et al., 2018, for a discussion of the relationship between transparency and decomposability). After this, the procedure started again for the next phrase, until all 40 idioms on any given list had been seen and rated. Within each list, idioms were presented in random order.

Participants were then asked to take the modified version of the Vocabulary Size Test. They were shown a word, followed by the same word in a short, neutral sentence (e.g. *refectory*: *We met in the refectory*), and given four possible meanings to choose from. A fifth “I don’t know option” was added, and participants were instructed to avoid guessing for words they didn’t know, but simply to choose “I don’t know” and move on. Items were presented in a fixed order, starting with more frequent and ending with less frequent words.

All data was collected using Qualtrics (www.qualtrics.com) to administer the survey, which took around 25 min on average to complete.

## Results

### Overall Rating Data as a Function of Age

For descriptive purposes, participants were divided into age brackets of approximately 10 years. Table 1 provides an overview of the idiom rating and vocabulary test data according to participant age. Overall familiarity was high (mean = 4.62/5, SD = 0.93), ranging by subjects from 2.9/5 to 5/5.

**Table 1:** Summary of familiarity and transparency ratings for all items, and vocabulary scores, divided by age group (means, with SDs in brackets); education level lists the number of participants in each age bracket at each level of education. Unknown items are those that scored 1 on the familiarity rating. False alarms are those which participants indicated had a different meaning to the one they initially thought

	Age bracket (N)						
	18–25 (48)	26–35 (37)	36–45 (38)	46–55 (40)	56–65 (43)	66–80 (31)	Total (237)
Familiarity (all items)	4.25 (1.26)	4.62 (0.94)	4.61 (0.88)	4.81 (0.71)	4.72 (0.76)	4.85 (0.58)	4.62 (0.93)
Unknown idioms	7.9%	3.5%	2.8%	2.3%	2.2%	1.2%	3.5%
Familiarity (known items)	4.53 (0.87)	4.76 (0.64)	4.72 (0.63)	4.90 (0.41)	4.80 (0.52)	4.90 (0.40)	4.76 (0.63)
False alarms (known items)	8.4%	4.5%	4.4%	2.9%	3.4%	2.0%	4.4%
Transparency (all items)	3.34 (1.31)	3.39 (1.26)	3.38 (1.27)	3.36 (1.31)	3.24 (1.30)	3.34 (1.35)	3.34 (1.30)
Transparency (known items)	3.48 (1.25)	3.46 (1.22)	3.43 (1.24)	3.40 (1.29)	3.28 (1.28)	3.36 (1.34)	3.40 (1.27)
Transparency (unknown items)	1.77 (0.91)	1.44 (0.67)	1.49 (0.80)	1.61 (0.96)	1.30 (0.57)	1.73 (0.96)	1.61 (0.86)
Transparency (false alarms)	1.95 (0.85)	2.16 (0.86)	2.18 (0.93)	1.84 (0.80)	2.44 (1.25)	1.96 (0.96)	2.08 (0.95)
Vocabulary (/50)	24.8 (8.2)	26.1 (8.7)	27.9 (10.0)	35.4 (7.5)	36.5 (8.3)	41.1 (6.4)	31.5 (10.1)
*Education level*							
Secondary school	15	6	9	10	16	9	65
Vocational qualification	2	6	8	7	7	8	38
Undergraduate degree	26	16	16	11	13	8	90
Postgraduate degree	5	7	4	10	7	5	38
Doctoral level	0	2	1	2	0	1	6

Prior to analysis all continuous variables (age, familiarity rating, transparency rating, vocabulary scores) were centred and scaled. Familiarity ratings were analysed using linear mixed effects models or, for binary response variables (such as known/unknown and false alarms), generalised linear mixed effects models with binomial distribution, in R (version 4.2.1) and RStudio version (2002.07.0) using the packages lme4 (version 1.1-30) and lmerTest (version 3.1-3). Models included age as a fixed effect, subjects and items as random effects, and by-item random slopes for the effect of age.

**Fig. 1:**
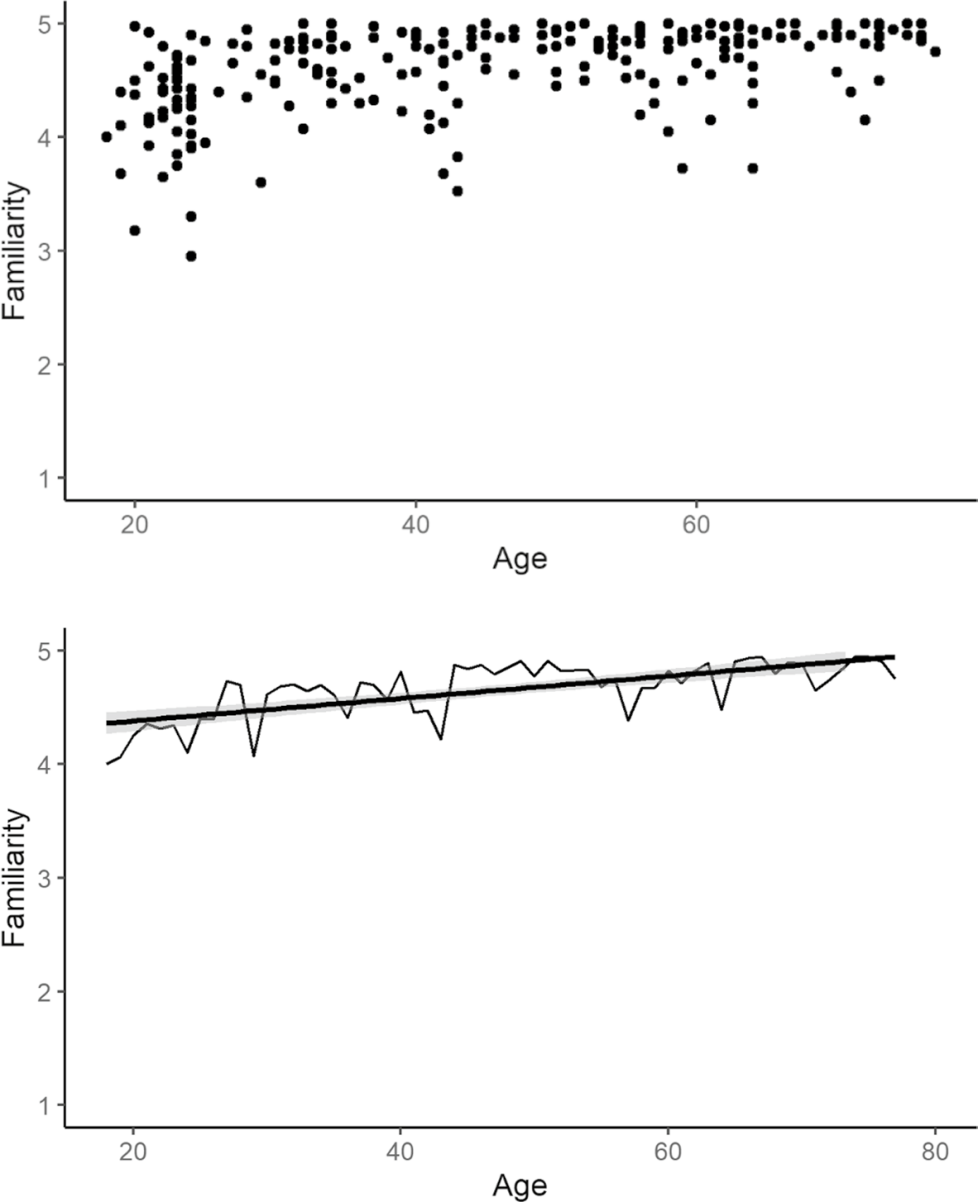
Average familiarity ratings as a function of age by subject (top) and grand mean by age (bottom)

Older participants showed greater familiarity with idioms overall (β = 0.19, *t* = 6.49, *p* < 0.001), had fewer unknown idioms (where familiarity = 1/5; β = 1.64, *z* = 4.28, *p* < 0.001) and fewer false alarms (thinking that an idiom had a different meaning than it did; β = 0.89, *z* = 4.94, *p* < 0.001). A model excluding unknown items also showed a significant effect of age (β = 0.13, *t* = 6.09, *p* < 0.001), confirming that the effect was not driven simply by older participants knowing more idioms, but that their knowledge of known idioms was in general better than younger participants. Figure 1 (top panel) also suggests that variability is higher amongst younger participants, while the bottom panel illustrates that although idiom knowledge is high across all participants, there continues to be a steady increase through the lifespan.

**Fig. 2:**
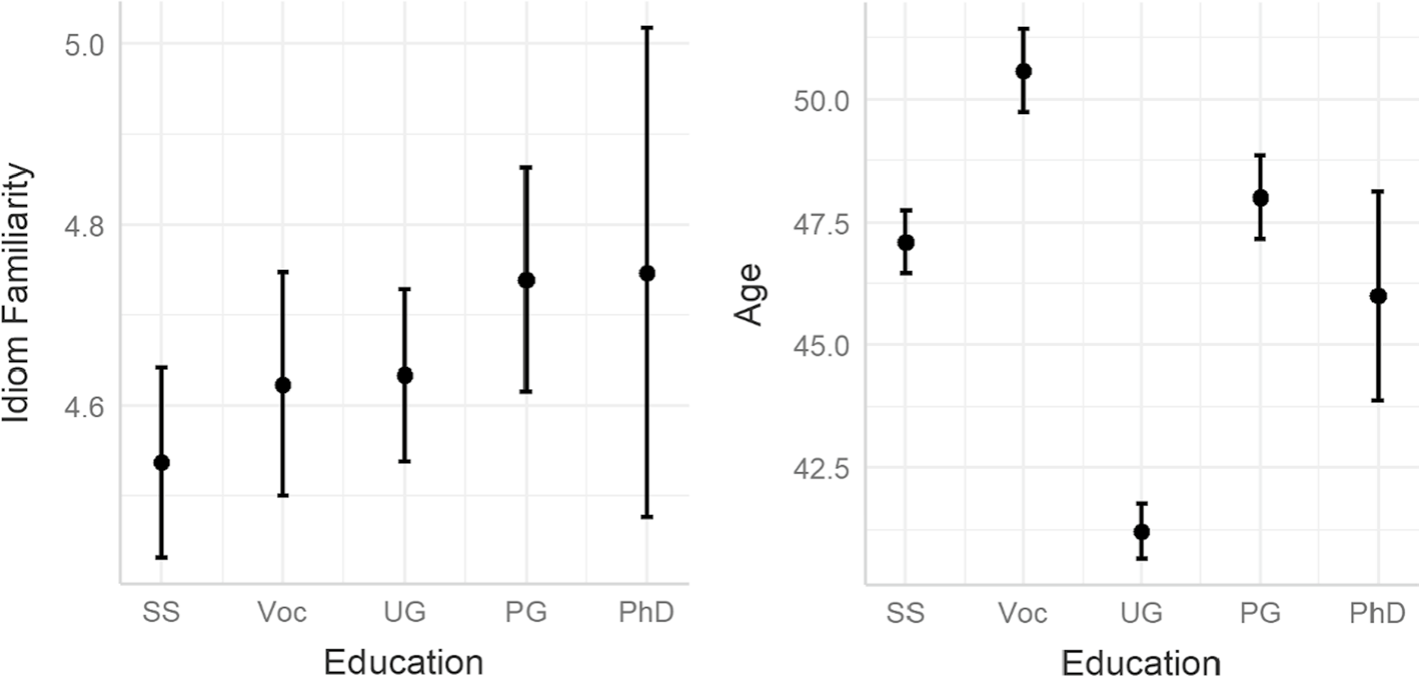
Idiom familiarity as a function of education level (left panel) and distribution of education levels by age (right panel). NB very few respondents (n = 6) were educated to PhD level, while all other levels had at least 38 participants. *SS* Secondary school; *Voc* Vocational qualification; *UG* undergraduate degree; *PG* postgraduate degree; *PhD* doctoral degree

Education level made an improvement to the model including age as a fixed effect (χ^2^ (4) = 10.19, *p* = 0.037), but no further improvement as an interaction term (χ^2^ (4) = 2.64, *p* = 0.620). Here, both variables had a positive effect on idiom familiarity. Figure 2 (left panel) demonstrates the effect of education on familiarity ratings, whilst the right panel demonstrates that education varied widely across participants in this study, not simply increasing by age. Whilst the most highly educated participants showed the highest ratings here, they also showed much greater variance than other groups. This may simply reflect the fact that only six out of 237 participants were educated to PhD level (around 2.5% of the participant pool), and these six participants ranged in age from 27 to 73.

**Fig. 3:**
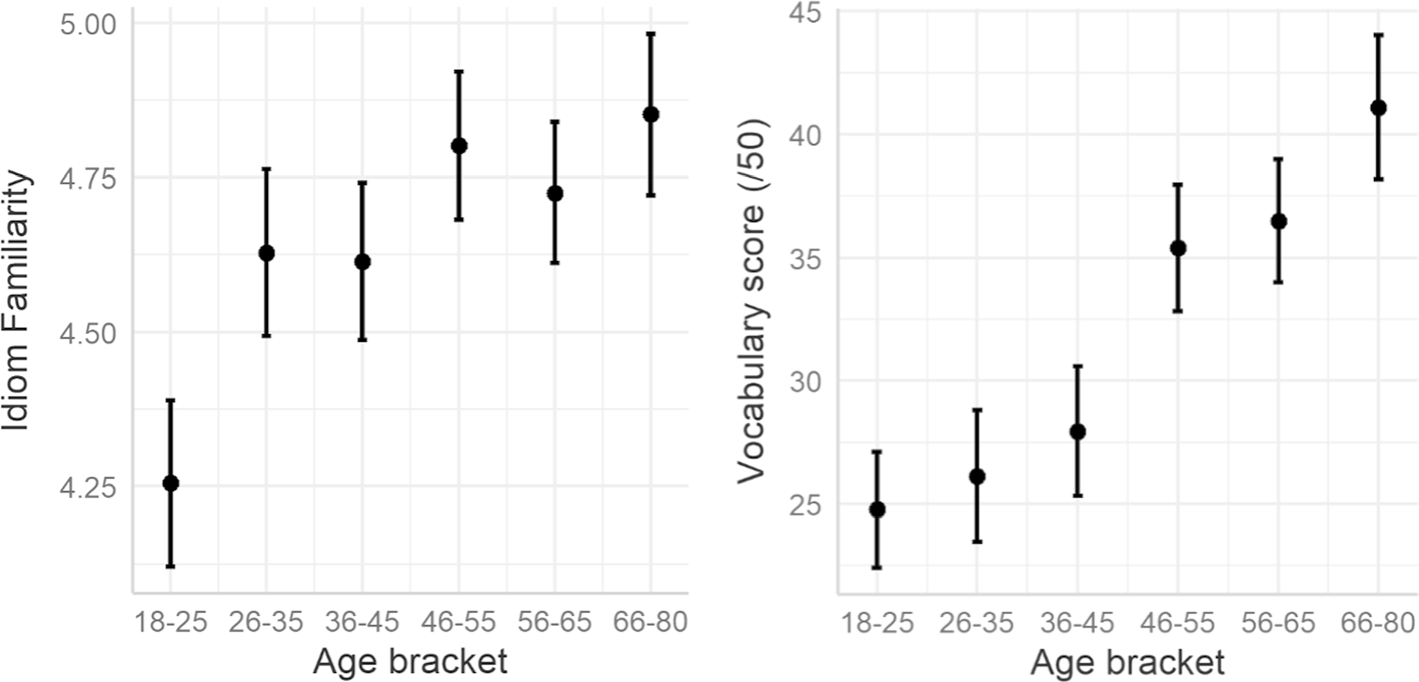
Idiom familiarity (left) and vocabulary knowledge (right) according to participant age bracket

Vocabulary scores were analysed using linear models with age as a fixed effect. There was a clear effect of age, with older participants demonstrating better single-word knowledge than younger participants (β = 0.56, *t* = 10.43, *p* < 0.001). Figure 3 compares the increase in idiom familiarity (left) and vocabulary knowledge (right), as a function of participant age. Education also improved the model for vocabulary as a fixed effect (χ^2^ (4) = 7.82, *p* < 0.001) but made no further improvement as an interaction with age (χ^2^ (4) = 1.06, *p* = 0.377). As with idiom familiarity, both age and education level made positive contributions to vocabulary knowledge.

Model comparison suggested that adding vocabulary scores along with age into the model for familiarity ratings made a significant improvement (χ^2^ (1) = 17.64, *p* < 0.001), but no further improvement was seen by including the interaction of age and vocabulary score (χ^2^ (1) = 2.23, *p* = 0.135). Despite the obvious correlation between age and vocabulary (*r* = 0.56, p < 0.001), the Variance Inflation Factor for each was below 2, suggesting no issues of collinearity in the model. Whilst vocabulary scores therefore increased with age, both appeared to make a contribution to idiom knowledge (i.e. older participants, and those with larger vocabularies had better idiom knowledge). With both age and vocabulary scores included in the model, education level made no further improvement (χ^2^ (4) = 4.82, *p* = 0.306).

Perceived transparency was not affected by age (β = − 0.02, *t* = -0.67, *p* = 0.501), vocabulary score (β = − 0.03, *t* = − 1.17, *p* = 0.245), or education level (all *t*s < 2.00, all *p*s > 0.05), but familiarity had a significant effect on transparency ratings, both for the whole dataset (β = 0.26, *t* = 27.20, *p* < 0.001) and for known items only (β = 0.29, *t* = 21.44, *p* < 0.001). Transparency was higher for known items than unknown (β = 0.73, *t* = 15.83, *p* < 0.001) and for correctly identified items than false alarms (β = 0.86, *t* = 25.98, *p* < 0.001).

**Fig. 4:**
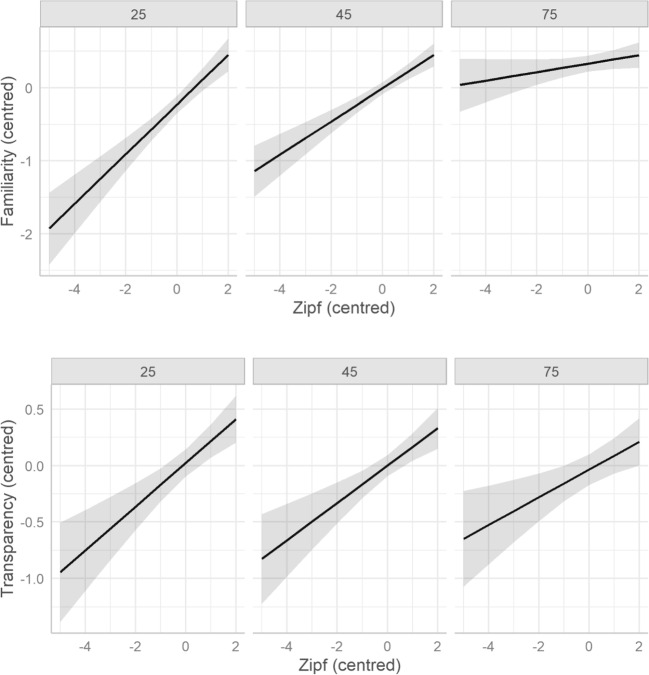
Predicted effects of frequency (on the Zipf scale, centred) on judgments of familiarity (top) and transparency (bottom), as a function of age (effects for participants at age 25, 45 and 75, from left to right)

Frequency was a significant predictor of familiarity (β = 0.14, *t* = 4.68, *p* < 0.001), and showed a significant interaction with age (β = − 0.01, *t* = − 5.12, *p* < 0.001). Figure 4 (top panel) demonstrates that the effects of frequency reduce as participants age. Higher frequency also contributed to higher transparency ratings (β = 0.15, *t* = 3.92, *p* < 0.001). Frequency also showed an interaction with transparency and age (β = − 0.02, *t* = − 2.25, *p* = 0.026), although the bottom panel of Fig. 4 suggests that this was less pronounced than the effect on familiarity.

### Individual Idiom Characteristics

Overall mean familiarity across the data was high at 4.62/5 (SD = 0.93). Within this, only one idiom (*a piece of cake* = “something very easy”) was rated as 5/5 by every participant (48 total ratings). A further 59 items were rated 4.9/5 or higher, and in total 156 out of the 200 items had average familiarity ratings of 4.5 or higher, suggesting that many of the idioms in the study were almost universally familiar to the participants regardless of age. Only 17 idioms were rated below 4/5 on average, and only 3 were rated below 3/5. These were one very old-fashioned idiom (*kick over the traces* = “act in a wild or insubordinate way”, mean = 2.19/5, SD = 1.59) and two much more modern idioms (*jump the shark* = “go beyond the realms of credibility”, mean = 1.85, SD = 1.37; and *jump the couch* = “act in an erratic and odd way”, mean = 1.70, SD = 1.15).

**Fig. 5:**
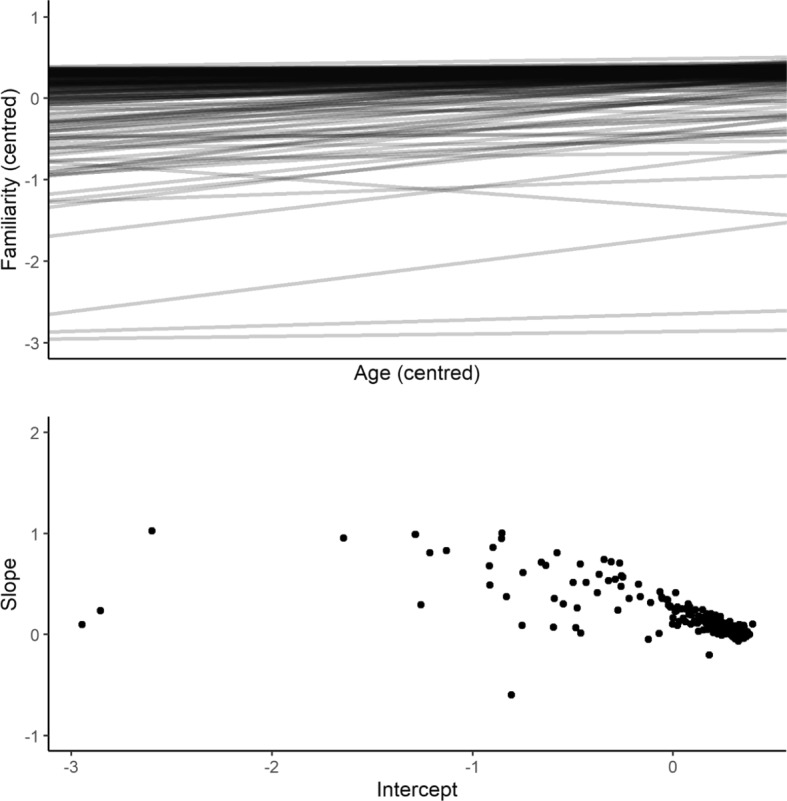
Model coefficients for by-item random slopes for the effect of age (top panel), and the relationship between slope and intercept across all items (bottom panel)

To better understand variation in the idioms used in this study, the random slopes for age were extracted from the model for familiarity. Of the 200 items in the study, 181 had positive slopes, whereby familiarity increased as a function of participant age. There was a strong negative correlation between slopes and intercepts (*r* = − 0.63, *p* < 0.001), and Fig. 5 (top panel) demonstrates a clear ceiling effect, whereby for a majority of items, familiarity was consistently high across all ages. Items with more positive slopes tended to have lower intercepts, suggesting that age effects were most pronounced for those items that were in general less familiar. The bottom panel of Fig. 5 also demonstrates this, with a clear cluster of items with negligible slopes and positive intercepts. In other words, for most items, familiarity started high and remained high across the ages sampled in this study.

Table 2 shows the number of items with slopes above and below the mean, and the number of items that fall within 1, 1.5, 2 and 2.5 SDs of this point. Overall, 169 items fall within 1 SD of the mean, and this figure rises to 192 for items falling within 2.5 SDs of the mean. Combined with the picture given by Fig. 4, this suggest that overall levels of familiarity are high and relatively homogenous in the data, with the age effect driven by a smaller subset of less familiar items (only 28 out of 200 items had random slope coefficients more than one SD above the mean, accounting for 14% of items in this study). The two items that were notable exceptions in that they saw substantial decreases in familiarity with age (more than 1.5 SDs below the mean) were both “modern” idioms (Carrol, 2022): *take one for the team* (= “incur personal injury for the benefit of a larger group”) and *break the internet* (= “generate massive attention online”), with absolute slope coefficients of − 0.20 and − 0.60, respectively.^2^ Although nineteen items had overall negative slopes, no other item had a slope coefficient smaller than − 0.06.

**Table 2:** Distribution of random slopes by item falling above and below the mid-point (mean = 0.19; SD = 0.25), and the distribution of items falling within different cut-offs

	Mean	1 SD	1.5 SDs	2 SDs	2.5 SDs
Above	64	36	43	50	57
Below	136	133	134	135	135
Total	200	169	177	185	192

A full set of idiom characteristics (means and SDs for familiarity, false alarm rate, transparency, frequency on the Zipf scale, item intercepts and item slope coefficients, presented for the data overall and sub-divided by age bracket) are available at https://osf.io/n2kfj/.

## Discussion

The data presented here are complementary to the data in Sprenger et al. (2019) for Dutch idioms. Idiom knowledge, measured here by perceived familiarity, number of unknown phrases and number of “false alarms” (where participants indicated familiarity with an idiom but subsequently discovered that they did not know the true meaning), increases with age. There is a marked jump from the 18–25 to the 26–35 age bracket (Fig. 3, left panel), followed by a steady increase, with relatively stable knowledge after around age 50. The overall pattern in Fig. 1 (bottom panel) is indeed more-or-less linear, but variability is much higher amongst younger participants (Fig. 1, top panel), as is the effect of corpus frequency on familiarity (Fig. 4, top panel). Both of these may be important factors in explaining the “lag” (relative to single word vocabulary, and similar to that seen in Sprenger et al., 2019) whereby idiom knowledge increases sharply from the 18–25 to the 26–35 age groups, then proceeds more linearly after that. Sprenger et al. (2019) suggested three possible reasons for this in their data: 1) the late development of figurative knowledge amongst children (discussed previously); 2) the fact that idioms often express complex or abstract ideas, hence may only be grasped fully later in adolescence/early-adulthood; or 3) that items used in their study may no longer be in common use. In the present study a wide range of items was purposefully included in the sample, and idiom knowledge was far from poor amongst the youngest age bracket (average familiarity of 4.25/5), hence the third of these seems unlikely to explain things on its own (although almost 8% of items in the study were unknown to participants in the lowest age bracket). The first two explanations seem plausible, and the lower familiarity scores for known items supports the idea that whilst younger speakers may have encountered many phrases enough to recognise them as idioms, their knowledge of the meaning (which may often come with particular connotations or pragmatic conditions) was still markedly lower than for older participants. Correspondingly, the increasing familiarity with age for items that were all known (to at least some degree) suggests deeper as well as broader knowledge amongst older participants. These results align well with the literature that suggest more entrenched idiom knowledge amongst older language users (Coane et al., 2014; Haeuser et al., 2021; Hyun et al., 2014).

There was no corresponding effect of age on transparency ratings, but familiarity with an idiom did increase the perceived transparency of a phrase. This was true both in terms of higher ratings for more familiar idioms, and higher ratings for known vs. unknown items (ratings for known items were roughly double those of unknown ones), as well as lower ratings for false alarms compared to known items. As with previous studies (e.g. Carrol et al., 2018), better knowledge of an idiom seems to inflate its perceived interpretability. The fact that we gave participants the meaning prior to asking for ratings of transparency also shows that this effect was not simply due to a lack of idiom knowledge, but implies that language users are more likely to see meaning connections in better known items in order to make sense of them (as seen in Keysar & Bly, 1995, where participants were given one of two conceptually opposite meanings for an unknown idiom, and subsequently rated the meaning they had learned as more transparent). Importantly, the lack of age effects here suggests that any semantic analysis that underpins these judgments is more or less stable across the lifespan.

Corresponding results for vocabulary also show a clear development with age. Brysbaert et al. (2016) suggested that vocabulary knowledge is relatively stable by age 20, with a steady increase of approximately two words per day up to around age 60. The present study supports this, with a broadly linear increase throughout the ages surveyed here, albeit with something of a leap from the 36–45 to 46–55 groups (Fig. 3, right panel). Lower frequency words may simply be encountered so rarely that a longer exposure to the language is required to master them, especially since studies of incidental learning suggest that multiple encounters are required before a form-meaning link can be developed (e.g. Jenkins et al., 1984 for first language reading; Pellicer-Sánchez, 2016 for second language reading). In comparison, “lower frequency” may mean something very different for idioms, and uncommon phrases may occur so rarely (relative to single words) that even longer is required for exposure to become likely, which may further explain the patterns seen for the youngest age bracket. Contrary to this idea (and the present data), the salience of idioms should make them more likely to be remembered after fewer encounters (Reuterskiöld & Van Lancker Sidtis, 2012), so further explanation is certainly necessary to fully explore this.

Educational level also affected vocabulary size (as in Brysbaert et al., 2016, and Keuleers et al., 2015, for Dutch vocabulary knowledge, where age, education and multilingualism were the most important factors affecting vocabulary size) as well as idiom knowledge, and further study into the effects of education may usefully explore exactly what areas might contribute to greater knowledge here (e.g. a PhD in a subject like literature may expose people to a greater range of idiomatic or figurative language than a PhD in the hard sciences, for example). However, whilst education level on its own predicted idiom knowledge, once age and single-word vocabulary size were factored in, education no longer made any contribution to how many idioms participants knew. Whilst education level therefore predicts vocabulary size, the latter seems to be a better indicator of how many idioms a speaker is likely to know (and how well).

Finally, the analysis of individual idioms demonstrated that a significant majority of phrases were generally very well-known across participants, with three-quarters of items rated above 4.5/5 on average. The proportion of unknown items was low (3.5%) overall, and the random slope analysis confirmed that the age effect seems to be limited to a relatively small set of less frequent idioms (around 14% of the items surveyed here). As addressed above, younger speakers may therefore simply not have encountered some more uncommon phrases, while older language users are more likely to have encountered them (probably multiple times) due to their greater exposure to the language. A point worth considering here is that younger speakers do presumably have their own set of idioms (and vocabulary/slang more generally), hence identifying and including such items in a study like this would undoubtedly present a very different picture. Whether such phrases can be considered as “idioms” more broadly (i.e. whether they make it into the language at large) is a separate question, but the underlying logic of how and why people develop knowledge of idioms (through exposure) would be the same, even for phrases that are relatively restricted and idiosyncratic. The main aim here was to investigate knowledge for the broad set of idioms that are generally seen as being a part of modern British English, but more group-specific phrases might well show less age-driven patterns.

Overall, the results support a view of idiom knowledge—and vocabulary knowledge more generally—as dynamic, with significant and ongoing development as a result of continual exposure to language throughout the lifespan. Whilst education contributes to both single-word vocabulary and idiom knowledge, age and vocabulary size combined are better predictors of how many idioms a person might know, and how well understood they might be. As a broad class of vocabulary item, idioms are well-known across native speakers, but both breadth and depth of knowledge increase with age, and less frequent examples may require much longer exposure to the language be assimilated into the typical adult native speaker vocabulary. Other factors not explored here—such as whether someone speaks other languages and to what level of proficiency—may also have important implications for both idiom and vocabulary knowledge, so may represent fruitful areas for further investigation.

## Data Availability

All data is available at https://osf.io/n2kfj/.

## Footnotes

1 The Zipf scale is a logarithmic scale reflecting relative frequency given the size of the corpus being used. A value of 1 represents 1 occurrence per 100 million words, 2 represents 10 occurrences per 100 million words, 3 represents 100 occurrences, and so on.).

2 Google Books Ngram viewer confirmed both of these as only gaining any level of widespread use post-2000.
